# A source-controlled data center network model

**DOI:** 10.1371/journal.pone.0173442

**Published:** 2017-03-22

**Authors:** Yang Yu, Mangui Liang, Zhe Wang

**Affiliations:** 1 Institute of Information Science, Beijing Jiaotong University, Beijing, People’s Republic of China; 2 Beijing Key Laboratory of Advanced Information Science and Network Technology, Beijing, People’s Republic of China; Southwest University, CHINA

## Abstract

The construction of data center network by applying SDN technology has become a hot research topic. The SDN architecture has innovatively separated the control plane from the data plane which makes the network more software-oriented and agile. Moreover, it provides virtual multi-tenancy, effective scheduling resources and centralized control strategies to meet the demand for cloud computing data center. However, the explosion of network information is facing severe challenges for SDN controller. The flow storage and lookup mechanisms based on TCAM device have led to the restriction of scalability, high cost and energy consumption. In view of this, a source-controlled data center network (SCDCN) model is proposed herein. The SCDCN model applies a new type of source routing address named the vector address (VA) as the packet-switching label. The VA completely defines the communication path and the data forwarding process can be finished solely relying on VA. There are four advantages in the SCDCN architecture. 1) The model adopts hierarchical multi-controllers and abstracts large-scale data center network into some small network domains that has solved the restriction for the processing ability of single controller and reduced the computational complexity. 2) Vector switches (VS) developed in the core network no longer apply TCAM for table storage and lookup that has significantly cut down the cost and complexity for switches. Meanwhile, the problem of scalability can be solved effectively. 3) The SCDCN model simplifies the establishment process for new flows and there is no need to download flow tables to VS. The amount of control signaling consumed when establishing new flows can be significantly decreased. 4) We design the VS on the NetFPGA platform. The statistical results show that the hardware resource consumption in a VS is about 27% of that in an OFS.

## Introduction

New techniques such as cloud computing and big data have promoted network applications, computing and storage resources to migrate to DCN [1]. By 2006 Google has had more than 450 thousand servers in its 30 DC. The number of servers in DCN for Microsoft and Yahoo also has reached to tens of thousands[2]. With the continuous expansions of the network scale, DC not only carries the traditional client/server applications but also carries new applications such as GFS and MapReduce[3]. This tendency has highlighted the DCN as the centralized status for information service infrastructure. In order to meet the challenges of new applications the DCN should satisfy the demands of high scalability and low configuration cost on the premise of green energy saving. The exploration of the new DCN architecture becomes hot in recent academic and industry research.

In face of new demands for DCN, the SDN technology has put forward the corresponding solutions[4]. The feature of single administrative entity in DCN makes it more suitable for centralization of management and control. The DCN is relatively independent of the Internet and there will be great possible reforms in network architecture for advantages of low cost and high efficiency. The technologies such as data identification, transmission and content location in SDN can well meet the new demand of technology development.

The paper is arranged as follows: In chapter 2, the background introduction is described. In chapter 3, the design of the SCDCN model is explained in detail. We conduct a series of experiments to study the advantages of the SCDCN model in chapter 4. In chapter 5, we summarize the paper and propose some schemes for further study.

## Problem description and background introduction

The employment of SDN technology in DCN has gained wide attention for its characteristics of centralized control and high scalability. However, the increasing volume in DCN has put forward severe challenges to SDN controller[5]. The flow-based SDN mechanism triggers the control plane more frequently comparing with traditional network, thus has led to greater overhead of bandwidth and delay for the communication between switches and the controller. Statistical studies have shown that there are a large amount of mice flows and their arrival and departure are so quick. About 80% flows that carry data volume are less than 10 KB, thus will lead greater overhead if all flows rely on one controller for scheduling and routing decisions. Meanwhile the network performance bottlenecks are prone to occur as the limitation of channel bandwidth and processing capacity of the controller.

The control function in SDN network is implemented by the controller which is mainly composed of a single centralized controller. This deployment strategy is simple and conforms to the characteristic of the logic centralized control. Yet there are scalability problems such as high decision-making cost, bad controllability and reliability when applying the scheme in large-scale data center network environment. Researchers have put forward a variety of solutions that are mainly summarized into three types: control plane multithreading technique, embedding the control logic into the data plane and distributed control plane solutions. Maestro and Beacon both belong to the control plane multithreading strategy. They initiate multiple threads to handle affairs so as to improve the control plane performance by increasing data throughputs and reducing events waiting time. However they can’t solve the scalability problems fundamentally for the reason that single controller still has upper limits of performance and available resources. DIFANE[6] and DevoFlow[7]both have alleviated the load of control plane by decentralizing part control functions so as to reduce the number of communication between control and data planes. DevoFlow has further considered how to reduce the collection overhead of network communication information. Yet the DIFANE requires authority switches in data plane that ordinary OpenFlow switches are incompetent. In addition the DevoFlow also needs to modify OpenFlow protocol and switches. These two methods are too difficult to actual network deployment and the schemes of embedding control logic into the data plane have violated the characteristic of the separation between data plane and control plane. As for distributed control plane solutions, HyperFlow and Onix[8] have improved performances of control plane by using multiple controllers or multiple controllers instances. They both apply network division tactics and each controller only maintains small-scale network that have alleviated the load of the single controller and solved the scalability in a certain extent. However they still face common problems of complexity of maintenance for global network view as to distributed control plane. Given this, the above schemes can’t well adapt to the large-scale DCN environment. The most effective way is to apply multiple controllers to multi-domain management[9].

**Fig 1:**
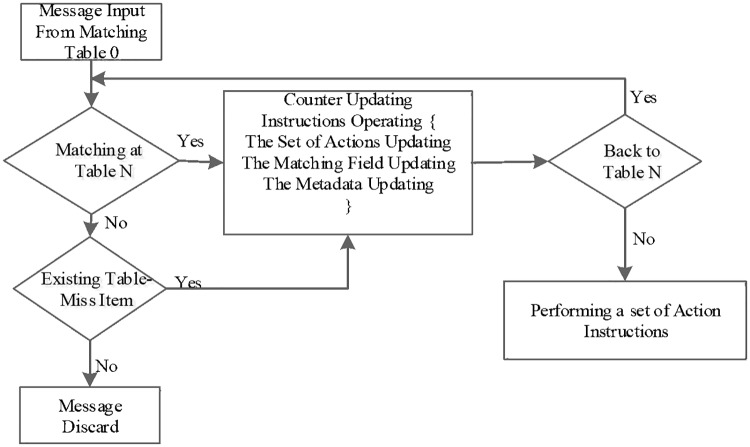
The message processing procedure in an OFS. There are one or more flow tables in an OFS, and the packet is matched against the flow entries to select a flow entry.

The OpenFlow protocol abstracts the data plane to a multistage-flow and table-driven forwarding model[10] (Fig 1). There are two important processes in data plane which are execution strategies and performing operations. The performing operations are invoked when taking the flow table (S1 Fig) lookup procedure. In case the process is complex, it can be realized through multiple iterations.

The core equipment in data plane is the OpenFlow Switch(OFS) which can achieve fast table lookup through the use of TCAM devices that enable high-speed and parallel search along with wildcard mask. However, the energy consumption and cost are too high and the storage capacity is also limited. An ordinary TCAM can only store thousands of flow table items[11].The above cases all limit the employment and extension of OFS in large-scale DCN. In addition, network flows show obvious elephant and mice flows that most flows are small and few large flows have taken up most of the data traffic. Many researchers have put forward some improved methods such as multistage flow table mapping algorithms[12],[13], flow table storage strategy combining with TACM,SRAM and NPU[14][15], the flow table storage optimization scheme based on resource reuse[13]. Yet again with the expanding scale and the increasing complexity of businesses in DCN, the rapid growth of flows along with the complexity of flow classifications all have increased the burden of table storage. The above-mentioned methods can’t solve the limitation of flow table storage fundamentally. The key to achieve superiorities of the SDN is to make feasible and efficient scheduling policy to various sizes of flows.

To sum up, using single controller for centralized network control and features of table lookup and storage all have limited the utilization of the SDN in modern large-scale DCN. In order to develop the DCN into a more energy-saving and flexible system, researchers should work out new methods and techniques in component-level, device-level and even network architecture level. In view of this, the paper puts forward the SCDCN model which introduces multi-controllers for multi-domain management. It adopts the VA as switching label in data plane which defines the communication path independently.

## The design and implementation of the SCDCN model

### The definition of VA

In view of elements to be encoded for data transmission, there are two kinds of coding methods in packets switching networks. One is to take the network nodes as elements addressed, and the representative is IP network. The other is to take the network links as elements addressed, and the representatives are MPLS and ATM networks. These two kinds of coding techniques can’t identify a communication path independently. For instance, a path is identified by both IP address and the routing table in routers in IP networks. In MPLS, a network path is identified by both switching label and the LFIB in routers. Similar to the MPLS, communication paths in OpenFlow networks are identified by both packets and flow tables in switches. Thus the forwarding process in data plane relies on signature strings in packets and flow table informations.

**Fig 2:**
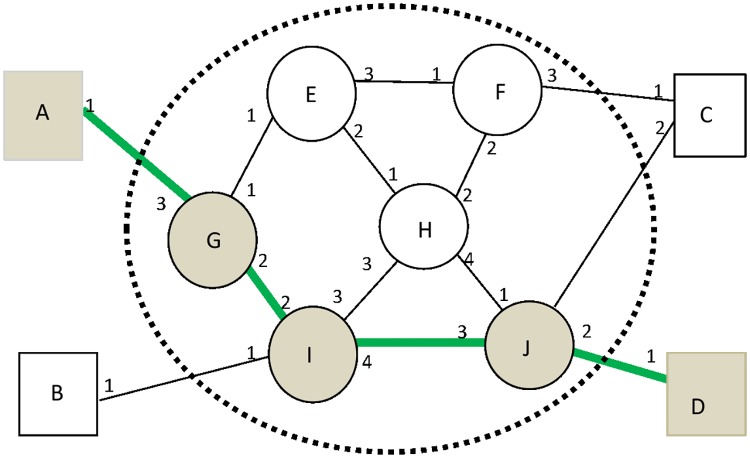
VA and the assignment of PI. Assuming the communication path from A to D is A->G->I.->J->D.

The vector address is a new type of coding technique[16][17]. It differs from the above-mentioned encoding types. The ports of switches are the elements of which this new kind of coding method is composed. A switch assigns each of its network ports a local port index(PI) sequentially. Thus we define the VA as a sequence of ordinal number of output ports in the communication path from the source node to the destination node[18]. The detailed description of VA is shown in Fig 2.

**Fig 3:**
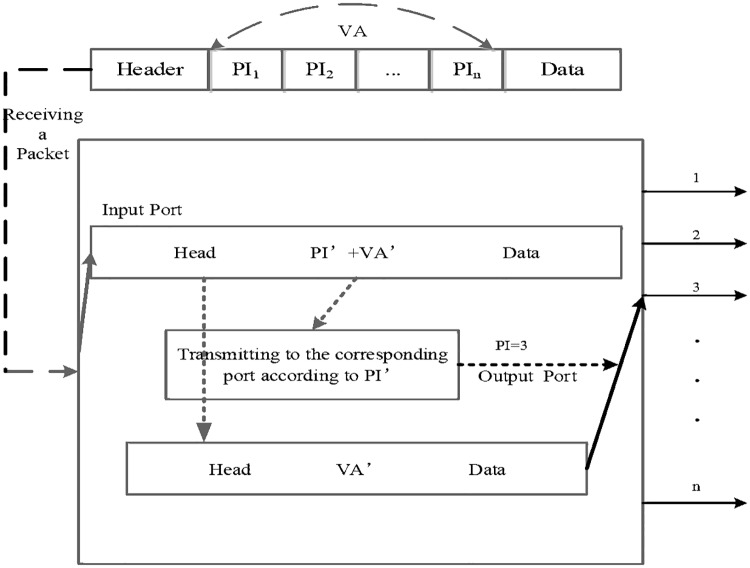
Switching operations in a VS. VA is a source routing address and the network can allocate a certain data forwarding routine solely relying on it.

**Fig 4:**
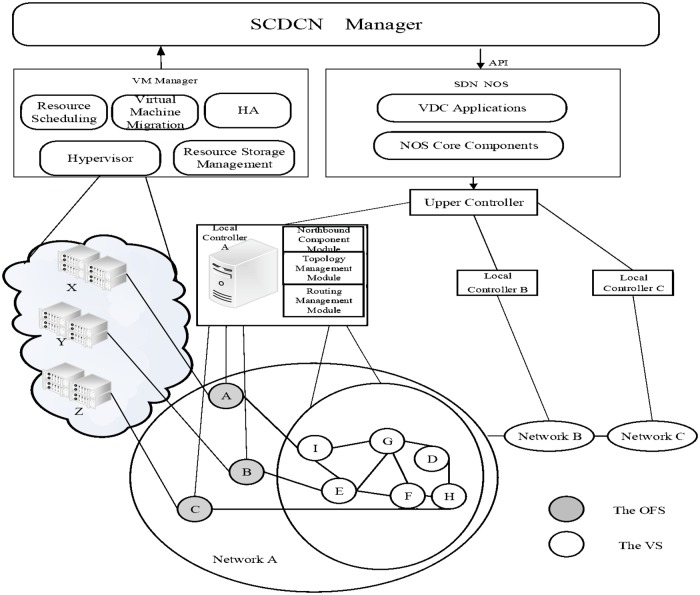
The architecture of the SCDCN model. The model makes network resources be unified with computing and storage resources and provides open interfaces for upper application by applying techniques of VA and SDN techniques.

There includes ES from A to D and VS from E to J successively (Fig 2). Suppose that the communication path from A to D is {A, G, I. J, D}, and that can be encoded as {1242} where figures 1, 2, 4 and 2 are corresponded to output port index of A, G, I and J respectively. The binary code of the VA is expressed as {1, 10, 100, 10}. Then the further result is integrated as {11010010} which can identify a communication path from A to D independently. As the address size of each switch differs from each other, it should be encoded beforehand in terms of the number of ports. The vector exchange is such a packet exchange process (Fig 3) which employs VA as data exchange address[19][20]. When a VS receives a packet, it first checks the packet and picks up the first element PI_i_ which is represented as PI’, and then encapsulates the VA’ into the header and sends the packet to the output port PI_i_ before the deletion of the PI_i_. By this analogy, the packet finally arrives at the destination.

Take V_ad_ for instance to explain the packet transmission process from A to D. As known that each VS is encoded in advance, eliminate PI’ of {1}, {10}, {100} and {10} respectively at node A, G, I and J. Then the VA is exhausted and the packet is sent to D finally. From the above process we can draw conclusions that the VS can fulfill the data forwarding process solely based on VA. Network can allocate a certain data forwarding routine according to VA and that means the VA is a source routing address. We apply this kind of source routing address as the packet switching label to data plane in DCN and adopt multiple controllers for multi-domain management in control plane that develops the SCDCN model.

### The SCDCN architecture

#### The overall design of the hierarchical control plane

**Fig 5:**
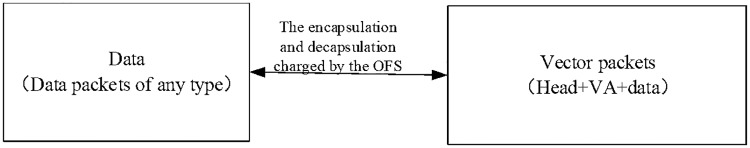
Packets encapsulation and decapsulation. The OFS has the function of encapsulation and decapsulation for packets.

**Fig 6:**
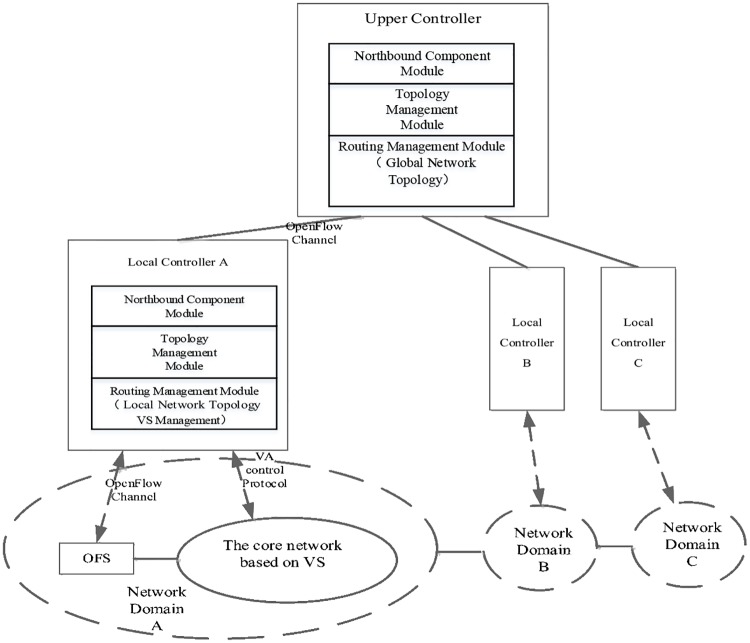
The control plane of the SCDCN model. The VS has simplified the model and only needs to cooperate to complete a few signal processing operation.

The applications of VA and SDN techniques in the new type of the DCN can well meet the demand of the management efficiency, high scalability and flexible resource scheduling. The SCDCN system is made up of three parts shown in Fig 4. The abbreviations listed in Table 1 are used to explain all notations and their definitions for Figs 4–6 throughout the paper.

**Table 1:** Abbreviations used in Figs 4–6.

Definition	Abbreviation	Definition	Abbreviation
Network Operating System	NOS	High Availability	HA
OpenFlow Switch	OFS	Virtual Machine	VM
Vector Switch^1^	VS	Vector Address^2^	VA

The adoption of abbreviations for certain keywords enables readers read and understand the paper more convenient and easier.

^1^ Vector switch, simply constructed and no need of table lookup and storage operations when conducting the data forwarding process.

^2^ Vector address, a new type of encoding technique and also a source routing address.

1. The SCDCN Manager
  The SCDCN manager is to realize the logic function of the DCN business for the whole multi-tenant. Meanwhile, it also combines SDN NOS and VM manager to unify control calculations and storage resources that can keep the synchronization schedule of calculation resources and network resources. There are correspondence mappings from each virtual machine for tenants to the network resource information which allows users master their own virtual network topology and make the scheduling and management by their own strategies.
2. The VM Manager
  The VM manager is consisted of resource scheduling, high available HA and virtual machine migration models. It is not only used to take charge of managements, calculations and storages for resources but also manage reports for creations and migrations for virtual machines. The VM manager supplies interfaces to SCDCN manager.
3. The SDN NOS
  The SDN NOS is the entity of the control plane that holds the view of the whole network. It is responsible for effective management and control for entire network resources.
  - The VS
    The OFS is only used in the edge of the SCDCN model. Flows become concentrated when come to the core network, the network shows the properties of high bandwidth, vase data volume and centralization. We take advantages of VS in high speed data forwarding process with which there is no need of table lookup and storage operation.
    In OpenFlow network, when there comes a new data flow, all OFS in the communication path should add the flow item. With the increase of network traffic, the limitation of storage capacity can’t satisfy the scalability in DCN. As for the SCDCN model, when a new flow arrives, only these two OFS in both network edges which act as gateways add the flow item respectively, while other VS in the communication path don’t involve any operations. Thus, there are great advantages in mass deployment of VS in new type of DCN.
  - The OFS
    The OFS is acted as gateway such as A, B and C in Fig 4 which is responsible for routing request and flow table lookup. In routing request process, when a new flow comes the OFS sends routing request signaling to controller for table updating. The OFS takes charge of the conversion operation between vector packets and any other types of packets such as MPLS and IP during the table lookup procedure.
    Comparing with the standard of OpenFlow, we only add the function of encapsulation and decapsulation operations for vector packets to the instruction fields in an OFS. The OFS carries out the flow table lookup operation as soon as the receipt of a new packet. If there is a match, it invokes the encapsulation of data packets by adding the head and VA to the original packet according to the instruction fields, and then it forwards packets to the corresponding output port for vector exchange. Otherwise the OFS will send a route request to local controller or upper controller. The controller carries out the routing computation and adds a new flow item consisted of VA information to the flow table for the subsequent data forwarding process. On the other hand, the OFS conducts table lookup operation after the receipt of a vector packet from the core network. Then the VA is run out, the OFS performs the vector decapsulation operation by cutting off the head and delivers the original packets to users. The specific conversion operations are shown in Fig 5.

#### The design of the SCDCN controller

A hierarchical control plane is proposed that applies multiple controllers to get better performance and scalability. A set of vector switches that a controller actually manages is defined as a network domain. And the controller is called local controller. An upper controller is referred to as the one which manages local controllers. A complete SCDCN architecture is composed of one or more network domains which are connected with each other. There are one or more local and upper controllers in control plane (Fig 6) and logic links are generated from the topology aggregation of links between network domains. We achieve the controller functionality through reforming the Ryu[21]. Controllers reuse part components of basic control function such as OpenFlow protocol analysis, Ryu components management, event dispatching and servers provided. Meanwhile the functions of VA collection and VS management are newly added. And on this basis the components modules in SCDCN control plane are designed which includes topology management, routing management and northbound component modules.

In topology management module there are four components. Topology discovery and synchronous component is used to collect topology information by distributing LLDP packets periodically and parse messages submitted by VS and local controller. Topology information component is applied to store the collected network information. Topology structure component is used to generate and store internal network topology view for routing function. Topology aggregation component is related to network aggregation and provides abstract network information to upper controller. In designing of the topology discovery, we apply link layer discovery protocol(LLDP) as the topology discovery protocol. LLDP provides a topology discovery method of relay type. The VS which supports LLDP agreement encapsulates its identity, address and port identifications and sends to directly connected network equipment. Neighbor devices receive and dump these messages so as to provide network link communication status to topology management module. In topology synchronization procedure, controllers achieve the network topology awareness and synchronization through active queries. An upper controller sends out Packet_OUT message carrying the LLDP packet periodically which adds a new information metadata TLV(Type, Length, Value). When a lower controller detects the new unit in a LLDP packet and identifies that the upper controller is performing the topology synchronization queries. Then it uploads the change of the network to assist the upper controller in updating the network view and fulfills the topology synchronization.

In routing management module there are three components which are routing, algorithm and upper table item analysis components. They are mainly used for data flow establishment, routing algorithm execution and VA collection.

The northbound component module connects with upper controllers which has three functions. First the component receives messages sent out from upper controllers for topology collection request, it does nothing but creates corresponding event and sends to other modules. Second it realizes the encapsulating functionality that sends the Packet_IN packet to upper controllers. Finally the module keeps the connection function with the upper controller. For example, the synchronous messages Echo sent from upper controller are used to check the connection status and the northbound component will return Echo Reply message response.

### The management mechanism of routing module

There are two important components in SCDCN routing process including path calculation and VA collection. Path selection is the core content in routing module. The corresponding VA can be collected by the selected routing path.

#### Routing algorithm

The vector network is expressed as VN = (G, L), G represents all network domains and L means inter-domain links. Each network domain represents as G = G(V,E^1^,E^2^). V is the node set and E^1^ is the internal network link set and expressed as e(u, v), u, v ∈V. E^2^ is the inter-domain link set and can be the same representing method, yet u or v is the port number. The source and destination node are expressed as s and d respectively. The routing path calculation operation can be expressed as follows.

1. The routing task is equivalent to the path finding operation in top network domain between top nodes s1 and d1 according to node s and d. It is assumed that G = G1 which represents top network domain. S1 is the top root node for s and the same representing method for d herein.
2. Run the intra-domain routing algorithm according to S and D. the input variables are the respective port sets W_s_^1^ and W_d_^1^ of source and destination node. The output are the top p optimal routing path set R(W_s_^1^, W_d_^1^) from S to D.
3. For each r belongs to R, r is represented as (w_s_^1^,v_1_,v_2_,…,v_n_,w_d_^1^). Each port of nodes on path r has a port information f and the expression is *f* = {B, D, N; *f*_i_|i∈[1,N]}. The port information for E_i_^2^ is *f*_i_ which is similar to *f*. The subport number is N. The port bandwidth B = ∑b_i_ which b_i_ is the bandwidth for *f*_i_. Meanwhile the parameter di is the delay variable and D = mind_i_.
4. Make G equals g_i_. S and D are the source and destination node sets for g_i_ respectively. And repeat step b) recursively.

In the above mathematical model, we have designed a cost modification multipath routing scheme as the intra-domain routing algorithm. The scheme is based on Dijkstra algorithm by constructing ridge terrain which has solved the distance problem between the routing paths. The detailed process is described as follows. First the controller calculates the shortest path according to the Dijkstra algorithm in its network domain. The shortest one refers to the path of least cost. Then before finding the second best path, we heighten the cost of nodes and links on the shortest path which has been calculated in the previous step to form terrain increment and run the Dijkstra algorithm again. The second shortest path is found and it must be the relative optimal path which has certain distance from the first shortest path. And by this analogy, the required number of paths is calculated.

#### The VA collection procedure

The VA collection operation is such a process that converts from routing paths to vector address. The specific steps are as follows. First the controller which directly controls the source node initiates a VA collection packet including port information of source and destination nodes. Meanwhile it writes vector address and reverse vector address of its own network domain into the packet according the routing path and deletes nodes information on the path. Then the controller transfers the packet to the boundary node of next network domain. The controller which manages the boundary node does the same operation as the former one, and so on till the packet reaches the network domain which the destination node belongs to. Finally the complete vector address and reverse vector address are delivered to the destination node. The destination node sends the VA acknowledgment packet to the source node according to the reverse vector address and the VA collection process is finished.

### The communication procedure of the SCDCN model

Take the information exchange scenario from X to Z for example to elaborate the communication process. We only compute one routing path and assume that the calculated path is X->A->I->E->F->H->C->Z herein. The specific operation steps are shown as below.

1. X wants to communicate with Z and sends a packet of any type named Pkt.
2. A performs the table lookup operation after the receipt of Pkt. If there is a match, then go to step ⑥, else A sends a routing request to the local controller.
3. The local controller checks whether A and C are in the same network domain according to the local topology information. If they are not, it sends the routing request to the upper controller which will start the routing module and calculate a routing path. Then it responses the routing request and downloads the new flow table item to both lower controllers, then these two local controllers issue the new item to A and C respectively. Otherwise go to step ④.
4. The local controller detects that A and C are in same domain and starts the routing module. The shortest path is calculated after running the intra-domain routing algorithm. Then the controller initiates the VA collection action and gets a VA labeled communication path. It subsequently issues the new flow table item to A and C respectively.
5. A receives the response from the local controller and adds the new item to its local flow table. Then it starts the packet forwarding operation.
6. A encapsulates the Pkt by adding the head and VA according to the matched flow table item, then the Pkt has been changed into the vector packet Pktva(Pktva = Head+VA+Pkt). Assuming that the routing path is A->I->E->F->H->C, A begins the vector exchange mode and sends the Pktva to I.
7. I receives the Pktva and continues to forward the packet to E in the same way. And by this analogy, with each jump for data forwarding the VA gets shorter and shorter and exhausted till it reaches C.
8. After the receipt of Pktva, C looks up the flow table and removes the head(the VA has been exhausted) to realize the decapsulation operation of the packet, Then C delivers the original packet to Z.
9. Z receives the packet and the session is over.

The transmission procedure from Z to X is the same as the above-mentioned only in the opposite direction. The mutual trust communication of any data type has been established afterwards.

## Experiments evaluations and validations

In the SCDCN model, the adoption of multi-controllers can well improve the processing capacity of controllers and solve limitations of control channel bandwidth. The routing efficiency has also been improved significantly. Meanwhile, we have optimized the data plane by employing vector exchange technology. Switches no longer need flow table storage and lookup operations. Thus has left out the devices of TCAM, RAM and NPU that has greatly reduced the complexity and resource consumption of switches. This improvement is of great significance in constructing new type of energy-saving DCN. We analyze the advantages of the SCDCN architecture herein.

### The routing comparison of the SCDCN and the OpenFlow network

This section we compute the statistics of signaling consumed in routing computation between the SCDCN model and the OpenFlow under the same conditions of routing information and network topology. We calculate the number of signaling under the circumstance of a known network topology. It is assumed that there are n nodes which the data flow would pass through.

In OpenFlow network, the number of signaling for the first OFS after the receipt of the packet is three which includes a PACKET-IN message, a Modify State Message and a PACKET-OUT message. The other n-1 OFS receive n-1 Modify State Messages in all. Thus the total number of control signaling is n+2. We can draw a conclusion that in OpenFlow network the number of signaling in routing computation process has a liner relationship with the path hop n.

In the SCDCN model, there are two conditions to discuss. One situation is that the two edge OFS are in the same domain. And the number of the signaling for the two edge OFS needed is four which includes a PACKET-IN message, two Modify State Messages and a PACKET-OUT message. The left n-2 VS along the routing path no longer need to update the flow table. In VA collection phase there are a VA collection packet and a VA acknowledgment packet. Thus the total number of signaling is six. The other situation is that the two edge OFS are not in the same domain. These two OFS still need four messages, meanwhile, the number of signaling for the upper controller is 3 (a PACKET-IN message, two Modify State Messages). Thus the number of signaling needed is 9 in all. We can come to a conclusion that the number of control signaling for routing computation and flow table updating in SCDCN is independent of the hop count. To sum up, the proportion of the number between the OpenFlow and the SCDCN is (n+2)/6 or (n+2)/9. In reference[22], authors have verified the conclusion that the maximum hop count in two-layer data center network is 6. We take the value of n for 6 and calculate that the average number of signaling in SCDCN accounts for about 80% of that in OpenFlow. The procedure of routing and flow table updating has been simplified in the SCDCN model. Meanwhile the cost and complexity for switches have been cut down significantly.

### The performance comparison of the VS and the OFS based on NetFPGA

**Fig 7:**
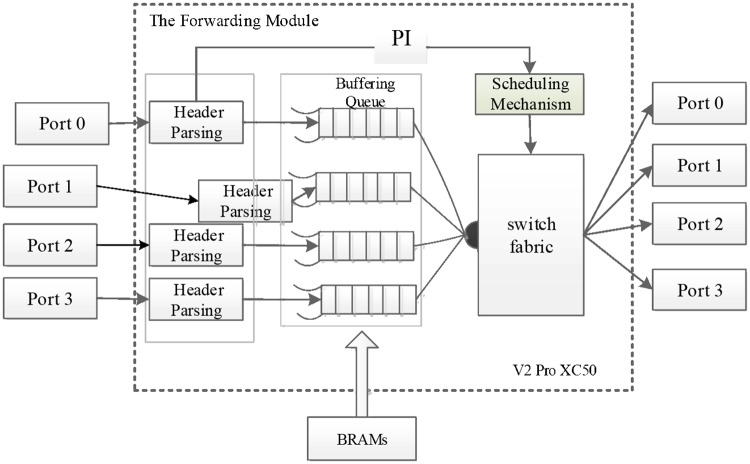
The structure of the VS based on NetFPGA. The packets are placed in buffing queues ranging from 0 to 3 based on off-chip BRAM without the use of off-chip SRAMs and DRAMs.

The NetFPGA is a low-cost and reusable hardware platform provided for researchers[23][24]. It allows researchers to establish Gb/s level high-performance network system model. The NetFPGA device introduces Xilinx Virtex-II Pro 50 as the main chip that contains 47232 LUTs and DFFs, 232 BRAMs and 23636 Slices. Meanwhile, there are JTAG test ports, 64MB DDR2 DRAMs and 4.5 MB SRAMs in onboard resources. As proposed in reference[25],[26], authors have developed an OpenFlow switch based on the NetFPGA. It employs two sets of flow table storage schemes which are the wildcard table lookup based on TCAM and the precise table lookup based on SRAM respectively. The TCAM based table lookup scheme adopts on-chip Xilinx SRL 16e that can realize 32 bit table items storage. On the other hand, the SRAM based table lookup scheme employs off-chip 4.5 MB SRAM which can store 32768 items. Furthermore the NetFPGA group has developed reference design models for gigabit Ethernet switch and IPv4 router. Based on the previous study and research, we have developed VS for the SCDCN model based on the NetFPGA. The specific packets forwarding procedure is shown in Fig 7.

When receives a packet, the VS first extracts the PI component for head parsing. The scheduling mechanism conducts the exchange array scheduling on the basis of PI. Then the packets are placed in the buffing queues ranging from 0 to 3 based on off-chip BRAM. Thus has not only solved the speed limitation for off-chip storage, but also has reduced the resource cost and consumption without the use of off-chip SRAMs and DRAMs.

We have made a detailed statistics about the hardware resources consumed from several aspects such as Slices, BRAMs and LUTs. Meanwhile, we conduct a set of experiments for performance comparisons among the VS, the OFS, the Ethernet switch and IPv4 router. The specific comparison results are listed in Table 2.

**Table 2:** Resource statistics for several kinds of switch hardware based on the NetFPGA.

	VS	OFS	IPv4 Router	Ethernet Switch
LUTs	3290	12691	17482	4750
DFFs	2583	8028	4136	1206
BRAMs^a^	38	20	24	106
Slices	1986	7134	--	--
Off-chip Memory^b^	None	DRAM,SRAM	DRAM, SRAM	--

Resource statistics can well reflect the utilization and consumption states of hardware resources for these switches.

^a^ BRAMs, on-chip memory implemented using FPGA cells.

^b^ Off-chip memory, off-chip memory latency is mainly determined by DRAM latency and memory bandwidth is determined by data transfer rate through the memory bus.

The results of statistical analysis indicate that there are great advantages for applying VA technology to large DCN.

1. The chip resources in a VS are consumed about 27% of that in an OFS.
2. Comparing with the OFS, there is no off-chip SRAMs in a VS. On the other hand, the OFS not only applies SRAMs for flow table storage but also needs the support of the PCI bus when updating a flow table. Thus each adjunction of a new table item consumes no less than 12 μs that has seriously cut down the rate for table updating operation.
3. The VS applies the chip BRAMs that the size of the packets buffer is only 40 KB. On the other hand, the OFS adopts the off-chip DRAM for packets buffering which has occupied the DRAM higher relatively.

### The performance analysis for hierarchical control architecture

The following series of experiments conducted are used to test the performance of hierarchical control structure applied to the large-scale DCN. We use the network simulation platform Mininet to generate a large number of virtual switches and compare the scheme with single centralized controller and distributed controller in respect of the average setup time of data flows for all nodes.

**Fig 8:**
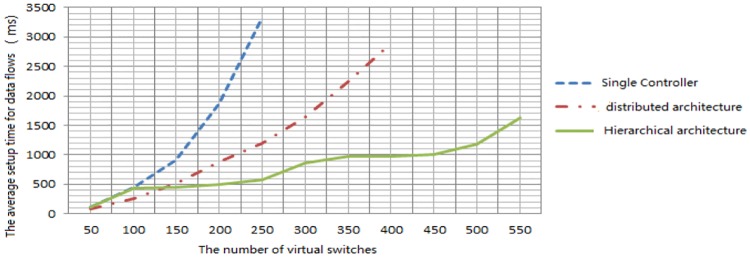
The average time of establishing data flows. The single centralized controller and distributed controller employ the native Ryu controller and ONOS controller respectively. There are no more than ten virtual switches in each domain and every five controllers are supervised by an upper controller.

We use the native Ryu controller and ONOS controller for single centralized controller and distributed controller respectively. In hierarchical multi-domain network architecture, the number of virtual switches in each network domain is limited to ten and every five controllers are controlled by an upper controller. As the network size becomes larger, the number of virtual switches continues to increase along with the layers in control plane. The comparisons of experiment results are shown in Fig 8.

We can draw conclusions that the establishing time of data flows in single centralized controller architecture increases rapidly with the expanding of network size. And that the setup time in distributed controller strategy has much smaller scope to rise on account of the application of multiple controllers. Yet the time required will also increase quickly. On the contrary, the increasing amplitude in hierarchical multi-domain controllers scheme is the smallest. The average setup time will be increased obviously only at the time of the increasement of layers in control plane.

From the experimental results we can get to the point that controllers in the same layer work in parallel in hierarchical control plane architecture and the average calculating time will not change sharply along with the expansion of data center network. The total establishing time for data flows is mainly effected by layers of controller plane. The hierarchical multi-controllers strategy has better performance comparing with other two kinds of control schemes. The hierarchical multi-controllers architecture can reduce decision-making costs for control plane and improve the network scalability and flexibility significantly.

## Conclusions and prospects

With the rapid development of information technology, there are many different data types that have involved explosive growth of information processing. To cope with features of high bandwidth and traffic in modern DCN, we employ multiple controllers for hierarchical multi-domain management based on OpenFlow technique that has not merely improved processing ability of controllers and routing efficiency but also reduced the computational complexity for control plane. Moreover, the VA technology is introduced as the switching label that has solved the constraints of flow table capacity and energy consumption. And on this basis we explore the SCDCN model. The core equipment in the SCDCN architecture is the VS and this innovation will greatly cut down the cost for maintenance and development in modern DCN. Furthermore, the amelioration will be in favor of fast deployment and implementation for new protocols and businesses in future DCN. Meanwhile, the open equipment and network will be an important research area in studying and building the future network architecture.

We have come to the conclusion that the new SCDCN architecture has advantages in energy saving and scalability through the theoretical analysis and experimental verification, yet there are still some aspects can be improved. First how to optimize the multiple routing protocol in hierarchical DCN is a key research direction in the future. Then in terms of reliability problems, such as how to appropriately backup and quickly switch to the standby controller is a research need to be further investigated next. Although the controller explored in the paper has realized the main function, there are still certain differences in functional effectiveness compared with the current mature commercial controllers. We will increase its usability in the follow-up work.

## Supporting information

S1 Fig The structural composition of flow table. A Flow table is a forwarding table which consists of multiple flow table items. Each item has its own execution action that is issued by the centralized controller.
(PDF)
