# Supplementary figures and images for: A source-controlled data center network model
Source: PLoS One. 2017 Mar 22;12(3):e0173442. doi: 10.1371/journal.pone.0173442 (PMC5362056; doi:10.1371/journal.pone.0173442)

**S1 Fig.** The structural composition of Flow table

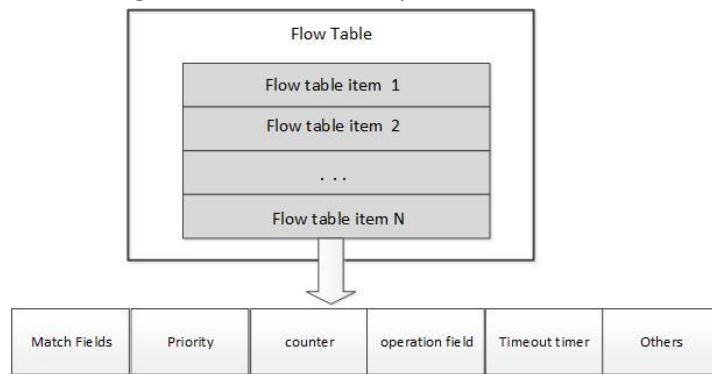

Supplement: S1 Fig — A Flow table is a forwarding table which consists of multiple flow table items. Each item has its own execution action that is issued by the centralized controller. (PDF) [file pone.0173442.s001.pdf]
